# The Counterintuitive Relationship between Telomerase Activity and Childhood Emotional Abuse: Culture and Complexity

**DOI:** 10.3390/ijerph18041619

**Published:** 2021-02-08

**Authors:** Clifton R. Emery, Qian-Wen Xie, Jessie S. M. Chan, Ling-Li Leng, Celia H. Y. Chan, Kwok-Fai So, Ang Li, Kevin K. T. Po, Zoe Chouliara, Cecilia Lai Wan Chan, Anna W. M. Choi, L. P. Yuen, Kam Shing Ku, Winnie Kung, Siu-Man Ng

**Affiliations:** 1 SWSA, University of Hong Kong, Pok Fu Lam, Hong Kong; linglileng@gmail.com (L.-L.L.); chancelia@hku.hk (C.H.Y.C.); cecichan@hku.hk (C.L.W.C.); 2 School of Public Affairs, Zhejiang University, Hangzhou 310027, China; xieqianwen377@163.com; 3 Department of Psychology, University of Hong Kong, Pok Fu Lam, Hong Kong; jessie.suet@gmail.com; 4 State Key Laboratory of Brain and Cognitive Sciences, University of Hong Kong, Pok Fu Lam, Hong Kong; hrmaskf@hku.hk (K.-F.S.); kevinktpo@gmail.com (K.K.T.P.); 5 Joint International Research Laboratory of CNS Regeneration Ministry of Education, Guangdong-Hong Kong-Macau Institute of CNS Regeneration, Jinan University, Guangzhou 510632, China; anglijnu@jnu.edu.cn; 6 Independent Practice, Edinburgh, Midlothian EH7, UK; zoe.choulara@outlook.com; 7 Department of Social and Behavioral Sciences, City University of Hong Kong, Kowloon, Hong Kong; ssanna@cityu.edu.hk; 8 International Association for Health and Yangsheng, 20 Venturi Rd., Happy Valley, Hong Kong; chinesemedicine9399@gmail.com; 9 Haven of Hope Haven of Hope Christian Service, 7 Haven of Hope Rd, Hong Kong; kukamshing@gmail.com; 10 Graduate School of Social Service, Fordham University, New York, NY 10023, USA; kung@fordham.edu

**Keywords:** child maltreatment, child emotional abuse, telomerase activity, long-term consequences, later adulthood, Chinese

## Abstract

BACKGROUND: A burgeoning literature has found relationships between telomere length, telomerase activity, and human health and longevity. Although some research links a history of childhood adversity with shortened telomere length, our review found no prior research on the relationship between child maltreatment history and telomerase activity in adulthood. We hypothesized a negative relationship between child maltreatment and telomerase activity and hypothesized that the association would be moderated by sex. METHODS: These relationships were tested on a sample of 262 Hong Kong Chinese adults (200 females versus 62 males) with mild to moderate depression. RESULTS: Counterintuitively, emotional abuse was positively associated with telomerase activity, while other maltreatment types were non-significant. The positive relationship between emotional abuse and telomerase activity was significantly moderated by the sex of the participant. CONCLUSIONS: We advance two possible explanations for this finding (1) a culturally informed resilience explanation and (2) a homeostatic complexity explanation. The two explanations are not mutually exclusive. This trial is registered under Hong Kong Clinical Trial Register number HKCTR-1929. SIGNIFICANCE STATEMENT: Emotional abuse was significantly positively associated with telomerase activity. There are at least two non-mutually exclusive explanations for the findings. Simply put, either (1) in the cultural context of Hong Kong emotional abuse was not a risk factor, and/or (2) the conceptualization of telomerase activity as a straightforward indicator of longevity is overly simplistic. The first story we might term a “resilience explanation” while the second we might call a “homeostatic complexity” story.

## 1. Introduction

The role of telomerase activity in the promotion of human health is complex and remains not well understood. Telomere length and telomerase activity are generally accepted biomarkers for cell longevity [1]. The activity of the enzyme telomerase replaces lost telomeric DNA, directly protecting telomeres and helping to maintain telomere length [2]. These telomeres are not fully replicated, and are hence shortened, each time cells divide [1].

Studies of mice engineered to block telomerase production show the mice age more rapidly and have lifespans shortened by 83% unless provided with a drug that stimulates telomerase production [3]. Less telomerase activity is associated with shortened lifespan. Correspondingly, more telomerase activity is associated with longer lifespan [4]. On the other hand, telomerase activity appears to play a role in conferring the cellular immortality that results in tumor development via unlimited cell division [5]. Around 90% of human tumors show telomerase activation [5]. A systematic review finds that telomerase activity is associated with psychological stress [6]. Women with high levels of chronic stress have been shown to have lower telomerase activity than controls [7].

Research on telomerase activity in humans is largely limited to western samples. Moreover, the research on telomerase activity contains apparent contradictions. For example, several studies have found a negative association between telomerase activity and chronic stress [6] and a positive association with meditation [8]. However, one study found a positive association between telomerase and psychological stress in the lab [9]. Addressing the potential bias in western studies of telomerase activity may shed light on the relationship between chronic psychological stress and telomerase activity because culturally rooted attributions about the stress and its source may influence the effect of stress on telomerase activity. This study is the first to examine the relationship between child maltreatment and telomerase activity. It begins to address both cultural and mental health limitations in the research literature on telomerase activity by examining the relationship between history of child maltreatment and telomerase activity in a sample of mild to moderately depressed Hong Kong Chinese adults.

### 1.1. Child Maltreatment and Its Impact

The World Health Organization (WHO) defines child maltreatment as “all forms of physical and/or emotional ill-treatment, sexual abuse, neglect or negligent treatment, or commercial or other exploitation, resulting in actual or potential harm to the child’s health, survival, development or dignity in the context of a relationship of responsibility, trust, or power” (9). Child maltreatment is usually classified on an empirical basis into one of four types: (a) physical abuse, (b) sexual abuse, (c) emotional abuse, and (d) neglect [10]. Globally, past 12-month prevalence estimates for each of these types of abuse are: 23% for physical abuse, 13% for sexual abuse, 36% for emotional abuse, and 16% for neglect [10]. In Hong Kong, lifetime prevalence estimates are 28.5% for physical abuse, 72% for emotional abuse, 0.9% for childhood sexual abuse, and 36% for neglect [11,12]. Child maltreatment is a “developmentally adverse interpersonal trauma” [13]. It interferes with healthy development processes including the development of emotional self-regulation [14], secure attachment relationships with caregivers [15], information processing [13], and self-attributions [16]. As seen in neuroimaging, maltreatment may alter brain structure and function, particularly in areas associated with attention and categorization of information [13]. In the longer term, maltreatment is associated with diabetes [13] (Ford, 2017), ischemic heart disease and cancer [17], posttraumatic stress disorder, depression, suicide attempts [18,19,20], addictions, early sexual debut [21,22] externalizing and internalizing behavior problems [23], and personality disorders [24,25].

Child maltreatment may leave a lasting imprint on an individual’s core physiology, resulting in a biological memory of the maltreatment [26]. An important new area of research on lasting physiological influences of maltreatment is in the area of cell aging. A growing body of research has linked child maltreatment history, including emotional neglect [27] (Rosser, 2014), with shorter telomere length [27,28,29,30]. However, to our knowledge no studies have examined the relationship between maltreatment and telomerase activity [26].

### 1.2. Matters of Sex and Culture

A growing literature examines the relationship between toxic stress and cortisol levels [31,32]. Chronically high levels of cortisol, (a hormone associated with stress generally and maltreatment specifically), are associated with shorter telomeres [33]. However, it is unclear whether there is a pathway from maltreatment to rapid cellular aging via telomerase activity because no studies have examined whether there is a link between maltreatment and telomerase activity. To date, research on stress and telomerase activity in humans has largely been reported based on Western samples, which limits the understanding of the role culture may play in the relationship between stress and telomerase activity. Telomerase plays a particularly important role for cells that must divide frequently, specifically in fetal somatic cells and in adult ovaries and testes [34]. However, neither mature spermatozoa nor oocytes normally show high telomerase activity [34]. Although there may be theoretical grounds in physiology to suggest sex differences in a relationship between maltreatment and telomerase activity, empirical findings of sex differences in the effects of maltreatment literature suggest the possibility of sex differences much more strongly. There is strong evidence to suggest that the effects of maltreatment differ by the sex of the child victim [35,36]. Telomerase activity has also been linked to estrogen in human ovary epithelium cells [37].

Cultural norms are critical yet typically underestimated in assessing the impact of child maltreatment. Although maltreatment increases the stress hormone cortisol [38], whether maltreatment leads to a chronic physiological imprint [39] is likely to be a function of child attributions about the maltreatment [40]. It is also likely related to the frequency, severity, and type of maltreatment, because abuse-specific internal attributions are consistently related to higher levels of psychopathology [41]. Victim attributions explaining why the abuse occurred are both critical to well-being and are profoundly influenced by cultural norms [42,43].

### 1.3. The Current Study

This paper reports the results of an exploratory study; the first that we know of to examine the relationship between a history of child maltreatment and telomerase activity. We examine this relationship in a sample of mild to moderately depressed Hong Kong Chinese adults who suffered poor sleep quality. We hypothesize that child maltreatment will be associated with lower levels of telomerase activity. The study also tests whether this relationship is moderated by the sex of the participant (victim). Depression is associated with telomerase activity [6,44] and child maltreatment [18,19,20]. For this reason, it is controlled in the analyses. The data used also selected for individuals with sleep disturbance. However, there was no relation between telomerase activity and the Pittsburgh Sleep Quality Index (PSQI) in the data, hence this variable was not controlled in the analyses.

## 2. Method

### 2.1. Study Design, Procedures, and Participants

The study design is cross-sectional analysis of baseline RCT data. The self-reported data come from baseline assessments of 262 mild to moderately depressed Hong Kong Chinese adults with sleep disturbance participating in a randomized clinical trial (RCT) conducted in Hong Kong in 2014 [45,46]. This RCT compared the effects of qigong exercise and Integrated Body Mind Spirit (IBMS) intervention, compared with being on a waiting list. CONSORT guidelines were followed when designing and reporting the RCT. The RCT reference population comprised 1,441 adults who were aged 18 to 55 years; who agreed to, and were available for, a blood test; did not have diagnosed medical conditions; and had not participated in qigong or IBMS training in the previous 6 months. Inclusion criteria for the study included a score ranging from 10 to 34 on the Center for Epidemiological Studies Depression Scale (CESD) and a score of 6 or higher on the Pittsburgh Sleep Quality Index (PSQI). Exclusion criteria included a history of psychosis or sleep disorders other than insomnia. Ethics approval (UW 13-485) was provided by the Institutional Review Boards of the University of Hong Kong and the West Hong Kong Island Cluster, Hong Kong Hospital Authority (see HKCTR-1929: http://www.hkclinicaltrials.com). All participants gave written informed consent before joining the study.

### 2.2. Measures

*Telomerase Activity*. Isolation of mononuclear cells was routinely performed as described previously [47]. Due to resource constraints only telomere activity (not length) was measured. In brief, 5 mL of the peripheral blood from each participant was collected in heparinized tubes, and subsequently mixed with an equal volume of the normal saline. Thereafter, 5 mL of the diluted heparinized blood samples were loaded on the top of a 5 mL Ficoll (GE Healthcare, Pittsburgh, PA, USA) and centrifuged (3000× *g*) at 4 °C for 10 min. The mononuclear layer was collected, added to 10 mL of the normal saline and centrifuged at 300× *g* at 4 °C for 5 min. The cell pellet was then mixed with a 200 μL lysis buffer, and incubated on ice for 30 min. The resulting lysate was centrifuged (16,000× *g*) at 4 °C for 20 min, and the supernatant was collected and stored at −80 °C until further processing using the subsequent telomerase activity assay with Telo TAGGG Telomerase PCR ELISA kit (Roche Molecular Biochemicals, Basel, Switzerland) following the manufacturer’s protocol [47].

In brief, the supernatants of the centrifuged sample lysates with telomere tandem repeats (TTAGGG) and telomerase were mixed with the reaction mixture with biotin-labelled primers. The sequence in the reaction mix was then amplified by PCR with a model TP600 thermal cycler (Takara, Tokyo, Japan). Part of the amplified product was denatured and hybridized to a telomeric repeat-specific detection probe which labelled with digoxigenin. The hybridized product was added into a streptavidin-coated microplate following by several washing procedures to allow the specific binding of it on the plate. The immobilized PCR product was then detected with an anti-digoxigenin-POD peroxidase-conjugated antibody. Lastly, the concentration of the probe was visualized by the enzymatic reaction of the peroxidase which oxidizes tetramethylbenzidine to form a colored reaction product on a 96-well microplate. After incubation, the optical density at 450nm was measured on a SpectraMax iD3 Multi-Mode Microplate Reader (Molecular Devices, San Jose, CA, USA). The value of telomerase activity was normalized to the 6 selected samples with moderate activities.

Child Maltreatment. The Childhood Trauma Questionnaire (CTQ) 28 item short form [48] was used to measure history of child maltreatment. The CTQ contains five subscales: emotional abuse (EA), emotional neglect (EN), physical abuse (PA), physical neglect (PN), and sexual abuse (SA). Each item is rated on a 5-point Likert-type scale with options ranging from never true (1) to very often true (5). To reduce collinearity in the models these scales were summed and combined into Physical Maltreatment (PA + PN), Emotional Maltreatment (EA + EN) and Sexual Abuse (SA) scales. The current sample reliability for Emotional Maltreatment was assessed by Cronbach’s alpha (α = 0.89). Current sample reliability for Physical Maltreatment was α = 0.80, and for Sexual Abuse was α = 0.88.

Depression Symptoms. Depression symptoms as measured by the CESD [49] were among the inclusion criteria for the study and may be correlated with telomerase activity and history of child maltreatment. Hence, CESD score was controlled in analyses. The mean of each CESD item was calculated and these were summed for all items. Current sample reliability for CESD was α = 0.71.

### 2.3. Statistical Analysis

**Figure 1:**
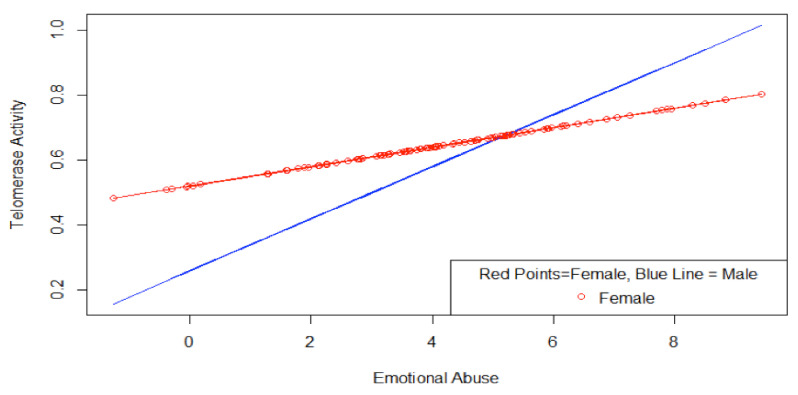
Telomerase Activity and Emotional Abuse.

Descriptive and Ordinary Least Squares (OLS) model statistics were calculated using the *Stata11* analysis package. Figure 1 was created using the *R* statistics package. The largest Variance Inflation Factors (VIFs) were 2.06 in Table 1, 1.92 in Table 2, and 5.55 in Table 3. Pregibon’s link test found no indication of nonlinearity in the OLS models in Table 1 (hat-squared = 3.7, *p* = 0.22), Table 2 (hat-squared = 3.7, *p* = 0.17), and Table 3 (hat-squared = 1.4, *p* = 0.22). Dfbeta statistics were calculated for Table 1, the first model in Table 2, and Table 3 in order to ascertain whether influential outliers biased coefficients. Twenty-four observations had an absolute Dfbeta score greater than $2/\sqrt{n}$ for one or more predictors. When these potentially influential observations for Table 1 were removed, the coefficient for emotional abuse and neglect remained positive but increased in size and significance (B_emotional_ = 0.01, *p* < 0.01). After influential observations were removed from the first model presented in Table 2, the coefficient for emotional abuse remained the same size but increased in significance (B_e-abuse_ = 0.02, *p* < 0.01). When influential observations were removed from the model presented in Table 3, the main effect for emotional abuse dropped to non-significance but did not change sign (B_e-abuse_ = 0.02, *p* = 0.11). However, in the same model the coefficients for male and the interaction term kept their sign and increased in size and significance (B_male_ = −0.41, *p* < 0.01; B_maleXabuse_ = 0.09, *p* < 0.01). As findings generally became stronger when influential outliers were removed, the tables provide coefficients and statistics with influential outliers included to ensure that findings presented in the tables were conservative. We also ran the models controlling for physical abuse and physical neglect separately. Neither of these items was significant when included separately in the models and the coefficients for emotional abuse do not change in sign or significance.

## 3. Results

### 3.1. Descriptive Participant Characteristics

The average age of the 262 participants was 55.2 for women (sd 9.3) and 57.8 for men (sd 10.1). Ages in the sample ranged from 21 to 81. Most of the sample was female (200 females versus 62 males). The mean telomerase activity level was 0.46 units (sd 0.38). With respect to maltreatment, means varied considerably by type. The mean score for physical abuse was 7.84 (sd 3.49). Means for other types were 9.62 (sd 4.01) for physical neglect, 12.92 (sd 5.25) for emotional neglect, 6.02 (sd 2.43) for sexual abuse, and 9.31 (sd 4.19) for emotional abuse. With respect to depression, the mean score on the CESD was 21.65 (sd 6.55).

#### Counterintuitive Finding for Emotional Maltreatment

Table 1 shows ordinary least squares regression findings for model 1, which examines the relationships between telomerase activity and emotional, physical, and sexual maltreatment. The first column shows unadjusted regression coefficients; the third column shows standardized regression coefficients. Rather than the hypothesized negative relationship between maltreatment and telomerase activity, the model showed a significant positive relationship between emotional maltreatment and telomerase activity (β = 0.20, *p* < 0.05). The relationship between CESD depression scores and telomerase activity was, as expected, negative (β = −0.13, *p* < 0.05). There were no main effects for age or sex.

### 3.2. Emotional Abuse Drives the Positive Association between Maltreatment and Telomerase

The unexpected finding was further explored to identify the nature of how emotional maltreatment was positively associated with telomerase activity. Table 2 presents the findings from two post hoc analyses. The main model examined whether emotional abuse, emotional neglect, or both were associated with telomerase activity, holding constant participants’ age, sex, CESD score, and other forms of maltreatment. Emotional neglect was not associated with telomerase activity (β = 0.06, *ns*), whereas emotional abuse showed significant positive association with telomerase activity (β = 0.18, *p* < 0.05). We further unpacked the unexpected finding for emotional abuse in the emotional abuse items models. These models show results of five different regressions. Each emotional abuse item was entered in a separate model controlling for age, sex, CESD score, and other types of maltreatment to examine which emotional abuse items appear to drive the unexpected positive finding. The positive association between emotional abuse and telomerase activity in the model is driven by two emotional abuse items on the CTQ: (1) People in my family said hurtful or insulting things to me (β = 0.16, *p* < 0.05), and (2) I felt that someone in my family hated me (β = 0.19, *p* < 0.05).

### 3.3. Sex Differences in the Emotional Abuse-Telomerase Relationship

Table 3 presents the findings for a test of whether the relationship between telomerase activity and the emotional abuse items varies by sex of the participant. The two emotional abuse items were combined (Cronbach’s α = 0.81) and interacted with male sex in a model controlling for age, CESD score and other forms of maltreatment. The male X emotional abuse items interaction term was significant (β = 0.29, *p* < 0.05) and the three terms (male sex, emotional abuse items, and male X emotional abuse items) were jointly significant (F = 3.95, *df* (3, 247), *p* < 0.01). When the interaction term is introduced the main effect for male sex, insignificant before, becomes significant (β = −0.28, *p* < 0.05). Considered together, the results suggest that for females, a one standard deviation increase in the emotional abuse items is associated with a 1/7th standard deviation increase in telomerase activity. In the interaction model, males have significantly lower baseline telomerase activity, but standard deviation increases in the emotional abuse items are associated with significantly and substantially larger standard deviation increases in telomerase activity (β = 0.14 + 0.29 = 0.43).

Figure 1 shows the estimated relationship between the emotional abuse items and telomerase activity for males and females. At the mean for the two emotional abuse items (4.20), the difference in contribution of the emotional abuse items between males (−0.26 + 0.08 × 4.2 = 0.076) and females (0.03 × 4.2 = 0.126) to units of telomerase activity is small because the positive interaction for males simply offsets the lower baseline for males. However, when levels of emotional abuse are high (e.g., 10) the effect size is much larger for males (−0.26 + 0.08 × 10 = 0.54) than for females (0.03 × 10 = 0.30). The difference in effect size for males and females at the highest level of emotional abuse (10) is 0.54−0.30 = 0.24 units. As the standard deviation of telomerase activity is 0.38, this is nearly a 2/3rds (63%) of a standard deviation difference in telomerase activity between males and females at the highest level of emotional abuse.

## 4. Discussion

Research on telomerase activity generally, and the relationship between child maltreatment and telomerase activity in particular, is new. In this exploratory study of the relationship between child maltreatment history and telomerase activity, we expected to find broadly negative relationships between all types of maltreatment and telomerase activity. Such findings would be consistent with accepted concepts of child maltreatment as a risk factor and telomerase activity as a protective factor for cellular aging. However, the findings are counterintuitive. Emotional abuse was significantly positively associated with telomerase activity. Non-emotional forms of maltreatment had negative but non-significant coefficients.

These findings are preliminary and require replication. Assuming future studies replicate our findings, there are at least two non-mutually exclusive explanations for the findings. Either (1) in the cultural context of Hong Kong Chinese several decades ago when maltreatment was more common, emotional abuse was not a risk factor, and/or (2) the conceptualization of telomerase activity as a straightforward indicator of longevity is overly simplistic. We propose two possible explanations. The first explanation we term the ‘resilience explanation’ while the second we term the ‘homeostatic complexity’ story.

### 4.1. Resilience Explanation

The idea of resilience stems from the recognition that despite exposure to terrible environmental stress some children unexpectedly thrive [50]. Understanding the conditions under which resilience is likely to occur has become a foundation for research on child maltreatment [51]. It is possible that, in the cultural context of Hong Kong Chinese roughly 20 to 40 years ago, believing that a family member hated one, or experiencing someone in the family saying hurtful things about one, was amenable to positive attributional revision. If the family member in question was the mother, at the time the child might have felt hurt, distressed, and even depressed. However, if the child later concluded that the mother was the proverbial ‘tiger mom’ [52], that the emotional abuse was carried out in the interest of teaching the child the cultural value of learning to “eat bitterness” [53] (i.e., swallow harsh circumstances and consequently ensure the child’s long-term success) the child might later interpret the abuse as at least being rooted in love. If the child’s abuse attribution later shifted from an internal attribution (e.g., self-blame) to an external attribution, research suggests this shift would be accompanied by a decline in psychopathology [41]. The consequences of such a shift might be further augmented by the attribution that not only was the abuse not the child’s own fault but occurred because the perpetrator cared about the child. Such a positive shift in abuse attribution might also cause a positive shift in self-attribution [16], resulting in increased self-esteem. It is also possible that in Hong Kong Chinese two-parent families, when one parent is the “tiger parent” [52], the other parent or perhaps a grandparent compensates to boost the child’s self-esteem. In either of these cases the emotional abuse, while so unhealthy for the child it cannot be condoned, could leave a positive physiological imprint on the adult that is consistent with the data. Moreover, as Straus has shown that child maltreatment appears to be decreasing over time [54], it is reasonable to conclude that levels of child maltreatment in Hong Kong 20 to 40 years ago were much higher than they are now. It is possible that cultural demands for humility and the ability to withstand humiliation among Hong Kong Chinese renders children raised in this manner better adapted to adulthood without revision of abuse attributions. It is also possible that those who achieve positive relationships in adulthood after suffering emotional abuse in childhood get a cognitive-emotional boost from the contrast that is reflected in telomerase activity. If the resilience explanation is correct, future child maltreatment research must investigate whether unique cultural adaptations may protect against expected relationships between chronic stress and telomerase activity.

### 4.2. Homeostatic Complexity Explanation

A fundamental assumption of this paper is that as a protective factor against telomere shortening [2], telomerase activity can be considered a biomarker for longevity [26]. In allowing cells to bypass the Hayflick limit [26,55], potentially indefinitely, telomerase activity may prevent cell senescence. Associations between low telomerase activity and diabetes mellitus [56] and between high telomerase activity and longevity in Ashkenazi centenarians [4], provide evidence that telomerase activity may translate into better health and even longevity. This hypothesis is further supported by experimental studies of accelerated aging in telomerase-deprived mice [3], and a negative association between chronic stress and telomerase activity in humans [7]. Counterintuitively, both acute psychological stress [9] and meditation [8] are positively associated with telomerase activity. Moreover, telomerase plays an important role when a mutated and dedifferentiated cell begins to rapidly divide. By conferring veritable cellular immortality via unchanged telomere length, telomerase activity has been identified as one of the key factors in the progression of cancer [5]. Simply put, conceptualization of telomerase activity as a biomarker indicating human longevity assumes that longevity at the cellular level translates to longevity at the organism level. Such an assumption obscures the fact that in some cases cell senescence is an absolute necessity for organism survival.

The means by which telomerase activity is tightly regulated to maintain homeostasis across functionally different cells throughout the organism is insufficiently understood [37]. Some cells that require frequent division (specifically in blastocysts and in the testes and ovaries of adults) show high levels of telomerase activity [34], while other cells (e.g., spermatozoa) do not. Likewise, the processes by which telomerase activity becomes dysregulated remain unclear. A better understanding of the mechanisms behind intra-organism variability in telomerase activity may allow us to more accurately predict the circumstances in which telomerase activity promotes organism longevity versus the circumstances in which telomerase activity precipitates organism mortality. Although findings on telomere length seem to fall more consistently on the side of shortened telomeres as a risk factor for mortality [57,58], the same logical concern applies for that case.

### 4.3. Sex Differences

Further research is needed to establish which of the two explanations for the findings is correct, and basic research is needed to better understand the mechanisms by which telomerase activity is regulated. Our findings also suggest that the relationship between emotional abuse and telomerase activity may be different by sex. Male participants had a significantly lower baseline level of telomerase activity and a steeper slope with respect to emotional abuse, while female participants had a significantly higher baseline and a shallower slope (see Figure 1). Further research should seek to replicate our findings and trace whether boys and girls root these differences in physiological response in the different emotional processing of emotional maltreatment. Of particular interest would be whether male/female differences in maltreatment attributions [41] lead to differences in physiological response to maltreatment.

### 4.4. Limitations

The study is exploratory and requires replication, particularly given the small sample size and number of covariates controlled. Future studies should completely control for participants mental health history. The cross-sectional design carried out on a non-random sample of Hong Kong Chinese adults limits the generalizability of the findings. For older adults, a childhood history of maltreatment is both remote yet potentially traumatic, increasing the probability of recall bias. Longitudinal research on a younger population could assist in further understanding how a relationship between maltreatment and telomerase activity unfolds chronologically. Further, the study requires replication as this study was conducted on a sample comprised of adults with mild to moderate levels of depression who suffered poor sleep quality. Unmeasured characteristics in the sample may have caused the relationship between emotional abuse and telomerase activity. Finally, due to resource constraints telomere length was not measured for this study. Examining the extent to which the findings for emotional abuse and telomerase activity translate through to telomere length would strengthen our understanding of this surprising finding.

## 5. Conclusions

Telomerase activity is an accepted biomarker for cell longevity specifically and human longevity generally [26]. The hypothesis of a negative association between history of child maltreatment and telomerase activity was not supported, as emotional abuse was positively associated with telomerase activity. Two non-mutually exclusive explanations were proposed. The resilience explanation suggests the positive relationship between emotional abuse and telomerase activity occurs because the life experiences and distinct cultural norms of Hong Kong Chinese render attributions for emotional abuse amenable to later positive revision. If our participants concluded as adults that they were emotionally abused and taught to “eat bitterness” [53] precisely because they were loved, the long-term imprint on physiology could enhance longevity at both cellular and organism levels. The homeostatic complexity explanation, however, calls into question whether telomerase activity can be used as a biomarker for organism longevity given the current state of knowledge. It may be that the use of telomerase activity as such a biomarker must be contingent on the cell’s function and level of development, as well as on an understanding of the processes by which telomerase activity is regulated to maintain optimal organism homeostasis. Longitudinal research is required across samples from different cultures and age groups to further test these explanations.

## Figures and Tables

**Table 1:** Telomerase Activity and Physical, Sexual, and Emotional Maltreatment for Model 1 (*n* = 254).

Model 1	Telomerase Activity		
Variable	*B*	*SE B*	*β*
Age	0.001	0.003	0.01
Male	0.02	0.058	0.03
Physical Maltreatment	−0.006	0.005	−0.10
Sexual Abuse	−0.003	0.01	−0.02
Emotional Maltreatment	0.009	0.004	0.20 *
Depression Symptoms (CESD)	−0.007	0.004	−0.13 *

Note: Ordinary least squares regression coefficients. *SE B* indicates standard error of regression coefficients. * *p* < 0.05.

**Table 2:** Telomerase Activity and Physical, Sexual, and Emotional Maltreatment (*n* = 254).

Model 2	Telomerase Activity					
	Main Model		Emotional Abuse Items Models			
Variable	*B*	*SE B*	*β*	*B*	*SE B*	*β*
Age	0.001	0.003	0.02			
Male	−0.02	0.06	−0.02			
Physical Maltreatment	−0.006	0.005	−0.10			
Sexual Abuse	−0.005	0.01	−0.03			
Emotional Neglect	0.004	0.006	0.06			
Emotional Abuse	0.02	0.008	0.18 *			
Called Stupid/Lazy				0.02	0.02	0.07
Parents wished never born				0.03	0.02	0.09
People in family said hurtful things				0.05	0.02	0.16 *
Felt s.o. in family hated me				0.06	0.02	0.19 *
Was emotionally abused				0.03	0.03	0.06
Depression Symptoms (CESD)	−0.007	0.004	−0.12 ^†^			

Note: s.o. Someone. Ordinary least squares regression coefficients. *SE B* indicates standard error of regression coefficients. ^†^ *p* < 0.10 * *p* < 0.05.

**Table 3:** Telomerase Activity and Emotional Abuse X Sex (*n* = 254).

Model 3	Telomerase Activity		
Variable	*B*	*SE B*	*β*
Age	0.001	0.003	0.03
Male	−0.26	0.13	−0.28 *
Hurtful things/hated me	0.03	0.02	0.14 ^†^
Male X hurtful/hated	0.05	0.03	0.29 *
Other Maltreatment	−0.004	0.004	−0.08
Depression Symptoms (CESD)	−0.006	0.004	−0.11 ^†^

Note: Ordinary least squares regression coefficients. *SE B* indicates standard error of regression coefficients. ^†^ *p* < 0.10 * *p* < 0.05.
